# Analysis of IFITM-IFITM Interactions by a Flow Cytometry-Based FRET Assay

**DOI:** 10.3390/ijms20163859

**Published:** 2019-08-08

**Authors:** Michael Winkler, Florian Wrensch, Pascale Bosch, Maike Knoth, Michael Schindler, Sabine Gärtner, Stefan Pöhlmann

**Affiliations:** 1 Infection Biology Unit, German Primate Center–Leibniz Institute for Primate Research, 37077 Göttingen, Germany; 2 Faculty of Biology and Psychology, University Göttingen, 37073 Göttingen, Germany; 3 Institute for Medical Virology and Epidemiology of Viral Diseases, University Hospital Tübingen, 72076 Tübingen, Germany

**Keywords:** IFITM, FRET, influenza virus

## Abstract

The interferon-induced transmembrane proteins 1–3 (IFITM1–3) inhibit host cell entry of several viruses. However, it is incompletely understood how IFITM1–3 exert antiviral activity. Two phenylalanine residues, F75 and F78, within the intramembrane domain 1 (IM1) were previously shown to be required for IFITM3/IFITM3 interactions and for inhibition of viral entry, suggesting that IFITM/IFITM interactions might be pivotal to antiviral activity. Here, we employed a fluorescence resonance energy transfer (FRET) assay to analyze IFITM/IFITM interactions. For assay calibration, we equipped two cytosolic, non-interacting proteins, super yellow fluorescent protein (SYFP) and super cyan fluorescent protein (SCFP), with signals that target proteins to membrane rafts and also analyzed a SCFP-SYFP fusion protein. This strategy allowed us to discriminate background signals resulting from colocalization of proteins at membrane subdomains from signals elicited by protein–protein interactions. Coexpression of IFITM1–3 and IFITM5 fused to fluorescent proteins elicited strong FRET signals, and mutation of F75 and F78 in IFITM3 (mutant IFITM3-FF) abrogated antiviral activity, as expected, but did not alter cellular localization and FRET signals. Moreover, IFITM3-FF co-immunoprecipitated efficiently with wild type (wt) IFITM3, lending further support to the finding that lack of antiviral activity of IFITM3-FF was not due to altered membrane targeting or abrogated IFITM3-IFITM3 interactions. Collectively, we report an assay that allows quantifying IFITM/IFITM interactions. Moreover, we confirm residues F75 and F78 as critical for antiviral activity but also show that these residues are dispensable for IFITM3 membrane localization and IFITM3/IFITM3 interactions.

## 1. Introduction

*The interferon-induced transmembrane protein* (*IFITM*) genes are highly conserved between vertebrates, and their gene products fulfill diverse functions. IFITM1-3 are antiviral effector proteins of the innate immune system and block entry of several viruses into target cells [1,2,3,4]. The analysis of *IFITM3* polymorphisms in human influenza patients [5], although not undisputed [6,7], as well as studies with *IFITM3* knock-out mice [5,8] provided evidence that IFITM3 plays an important role in the defense against influenza virus infection. Moreover, further studies showed that IFITM3 might also contribute to innate defenses against other viruses, including human immunodeficiency virus (HIV) [9,10,11,12], alpha- [13,14], filo- [3,15], corona- and flaviviruses [16,17,18]. It is noteworthy that IFITMs can also be beneficial for certain viruses. Herpesviruses can use IFITMs to ensure release of progeny particles from infected cells [19], and the human coronavirus OC43 was shown to employ IFITM proteins for host cells entry [20,21]. These observations indicate that IFITM1-3 operate in pathways that can positively and negatively regulate virus infection. Therefore, understanding how IFITM proteins modulate infection might provide a basis for novel strategies for antiviral intervention.

IFITMs are believed to block fusion of enveloped viruses with target cells at the stage of hemifusion and/or fusion pore formation by reducing membrane fluidity or by increasing spontaneous positive curvature of the outer membrane leaflet [22,23]. Amini-Bavil-Olyaee and colleagues suggested that this might be due to IFITM-dependent alteration of intracellular cholesterol transport [24]. However, these results are not undisputed [15,22,25]. Alternatively, the particular membrane topology of IFITMs may increase positive curvature [23], and this property might be augmented by IFITM/IFITM interactions. Membrane association of IFITM proteins is facilitated by intramembrane domain 1 (IM1) [26], which is palmitoylated, and this modification is required for restriction of viruses [27,28,29]. Mutagenic analysis of IFITM3 revealed that F75 and F78 within IM1 are required for blockade of virus entry and for IFITM3/IFITM3 interactions [26], suggesting that the two processes might be linked. However, these results await confirmation, and it is incompletely understood whether mutation of these residues impacts biological properties of IFITM proteins other than self-association and inhibition of virus entry.

Here, we used a flow cytometry-based fluorescence resonance energy transfer (FACS-FRET) assay [30] to analyze interactions of IFITM proteins and correlated them with antiviral activity. We provide evidence for extensive homo- and heteromultimerzation of IFITM proteins and confirm that residues F75 and F78 are important for antiviral activity. However, mutation of these residues was compatible with normal IFITM3 localization and IFITM3/IFITM3 interactions, indicating that F75 and F78 impact antiviral activity by a so far unknown mechanism.

## 2. Results

### 2.1. Establishment of FRET Measurements by Targeting of Non-Interacting, Cytosolic Proteins to Membranes

For two proteins which can freely diffuse in the cytosol, a robust FRET signal can be considered an indicator of protein–protein interactions. IFITM proteins are localized to membranes and measurement of robust FRET-signals at the plasma membrane has been reported before [30,31,32,33]. However, negative controls used in these experiments were mainly unrelated membrane localized host-cell proteins, and the FRET-pair employed in these studies, enhanced cyan fluorescent protein (eCFP)/enhanced yellow fluorescent protein (eYFP), has certain disadvantages such as residual dimerization capabilities, which limit their application in FRET measurements [34]. Therefore, we first aimed to establish a set of negative controls, firmly allowing us to exclude false-positive FRET signals arising from targeting of proteins to overlapping membrane subcompartments. In addition, we based our analysis on the improved FRET pair super cyan fluorescent protein (SCFP) and super yellow fluorescent protein (SYFP) [35] to overcome limitations associated with the standard eCFP/eYFP combination. 

**Figure 1:**
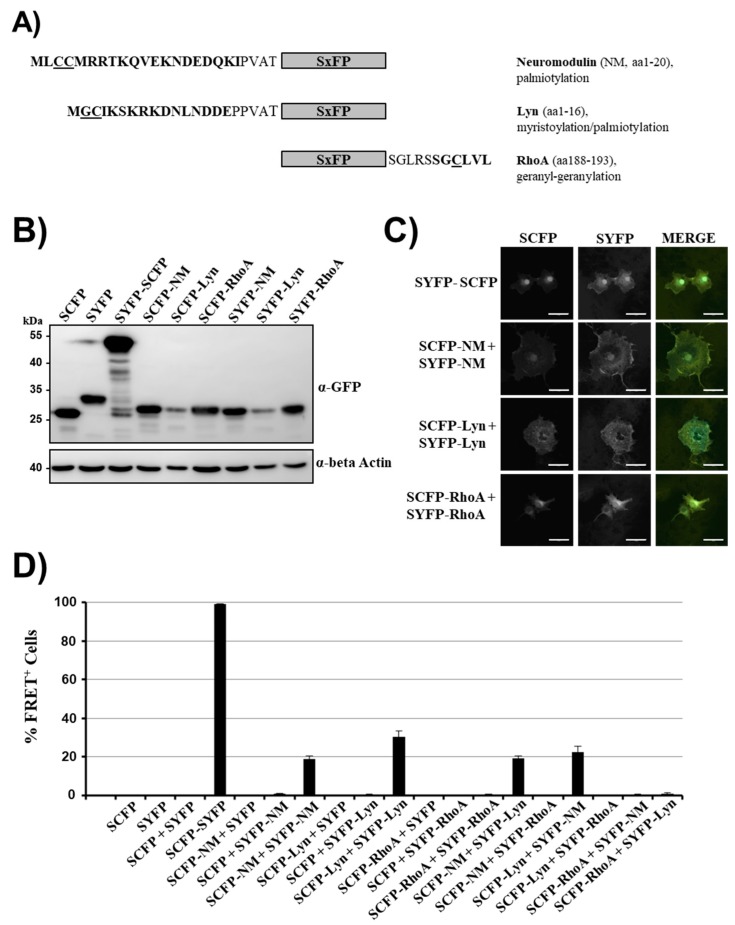
Membrane localization of non-interacting proteins can be sufficient for fluorescence resonance energy transfer (FRET). (**A**) Schematic presentation of super yellow fluorescent protein (SYFP) and super cyan fluorescent protein (SCFP) fusion proteins (SxFP) containing membrane targeting signals. The underlying cellular proteins, the relevant amino acid sequences and their respective modification for membrane targeting are indicated. (**B**) For analysis of expression of fusion proteins, 293T cells were transfected with the indicated plasmids and protein expression analyzed by Western blot, employing an anti-green fluorescent protein (GFP) antibody. Staining with anti-β-actin antibody served as loading control. Similar results were obtained in two separate experiments. (**C**) Immunofluorescence analysis of SxFP fusion proteins. Plasmids were transfected into COS-7 cells and protein expression was analyzed by confocal microscopy. The results were confirmed in two separate experiments. Scale bars indicate 50 µm. (**D**) 293T cells were transiently transfected with the indicated plasmid combinations and analyzed by FACS-FRET assay at 48 h post transfection. The results of a single experiment performed with triplicate samples are shown. Error bars indicate standard deviation (SD). The results are representative of 2–4 separate experiments. See Supplementary Figure S2 for representative primary FRET/CFP plots.

We fused the following membrane targeting signals to SCFP and SYFP, respectively: The N-terminal sequence of neuromodulin (NM, GAP-43), which targets the protein to lipid rafts within the plasma membrane due to palmitoylation of cysteines 3 and 4 [36,37] (Figure 1A) and the N-terminal peptide of the kinase Lyn, which is targeted to the plasma membrane by myristoylation of glycine 2 and is preferentially localized to lipid rafts due to palmitoylation of cysteine 3 [38]. Finally, the CAAX box at the C-terminus of RhoA was used, which is targeted to caveolae in the plasma membrane [39] due to geranyl-geranylation of a cysteine residue [40]. All SCFP and SYFP fusion proteins were efficiently expressed in transfected 293T cells and exhibited the expected molecular weights (Figure 1B, the presence of additional C-terminal amino acids derived from the polylinker region of pSYFP-C1 explains the larger size of SYFP as compared to SCFP, derived from pSCFP-N1). Expression levels were similar for all fusion proteins with the exception of SxFP-Lyn, which showed reduced expression levels (Figure 1B). The presence of membrane targeting domains resulted in pronounced membrane localization of the modified proteins relative to unmodified SCFP and SYFP (Figure 1C). Both SxFP-NM and SxFP-Lyn showed plasma membrane staining with some intracellular staining, whereas SxFP-RhoA showed a more pronounced staining of intracellular membranes with detectable staining at the plasma membrane (Figure 1C). The fusion protein SYFP-SCFP exhibited diffuse staining in the cytoplasm with strong accumulation in the nucleus, as expected [41]. 

Analysis of the proteins in the FACS-FRET assay (Supplementary Figure S1) revealed no signal upon coexpression of unmodified SCFP and SYFP while fusing both proteins resulted in a strong signal (Figure 1D, Supplementary Figure S2), as expected. When SCFP and SYFP were equipped with membrane targeting signals of neuromodulin and Lyn, low FRET signals were detected, while membrane targeting via the RhoA CAAX box did not result in FRET (Figure 1D, Supplementary Figure S2). Moreover, coexpression of SCFP and SYFP harboring different membrane targeting domains resulted in FRET when combinations of neuromodulin and Lyn membrane targeting signals were tested (Figure 1D, Supplementary Figure S2). In contrast, combinations in which one partner harbored RhoA CAAX while the other contained membrane targeting signals of neuromodulin or Lyn did not result in FRET (Figure 1D, Supplementary Figure S2). Thus, targeting of non-interacting, cytosolic proteins to membranes, potentially to specific membrane subcompartments, was sufficient to induce modest FRET signals in our assay. This may not be surprising, given that, for instance, certain lipid rafts can have a diameter of less than 20 nm [42]. Collectively, we were able to identify false-positive FRET signals of membrane targeted proteins in our FACS-FRET assay. These signals were modest and, based on the strong signal measured for the SCFP-SYFP fusion protein, should be markedly amplified by protein–protein interactions. 

### 2.2. Homotypic and Heterotypic Interactions between IFITM Proteins

**Figure 2:**
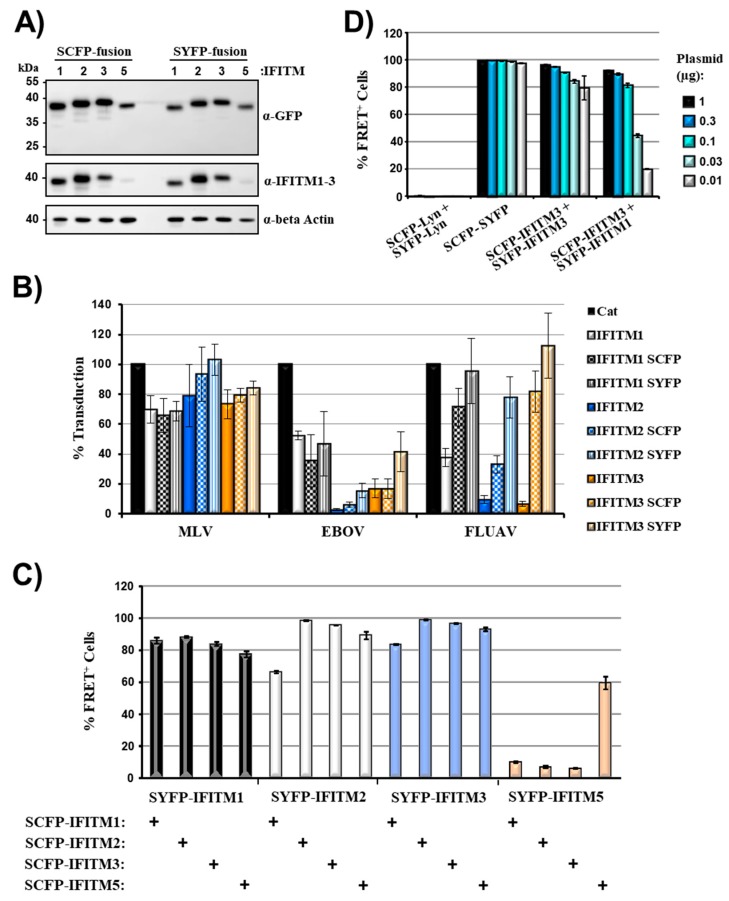
Robust fluorescence resonance energy transfer (FRET) upon coexpression of interferon-induced transmembrane protein 5 (IFITM5) with IFITM1-3. (**A**) For analysis of expression of IFITM fusion proteins, 293T cells were transfected with the indicated plasmids and protein expression analyzed by Western blot, employing anti-GFP (upper panel) or a mixture of anti-IFITM1 and 2/3 (middle panel) antibodies. The detection of β-actin (lower panel) served as loading control. Similar results were obtained in a separate experiment. (**B**) 293T target cells were transduced with vectors encoding the indicated IFITM proteins or chloramphenicol acetyltransferase (Cat) as control. Subsequently, the cells were transduced with infectivity normalized murine leukemia virus (MLV) vectors encoding luciferase and pseudotyped with MLV glycoprotein (MLV-Env), influenza A virus hemagglutinin and neuraminidase (FLUAV-HA/NA) or Ebola virus glycoprotein (EBOV-GP). Luciferase activities in cell lysates were determined at 72 h post transduction. Results are presented as % transduction efficiency of cat-expressing cells which was set as 100% and are representative of four individual experiments. Error bars indicate SD. (**C**) 293T cells were transiently transfected with the indicated plasmid combinations and analyzed by FACS-FRET assay. Controls are not shown but were similar to those shown in Figure 1D. The results of a single experiment performed with triplicate samples are presented and were confirmed in a separate experiment. Error bars indicate SD. (**D**) 293T cells were transiently transfected with the indicated plasmid combinations and analyzed by FACS-FRET assay at 48 h post transfection. Plasmids were used at the indicated amounts and total DNA amount was kept constant by filling in empty pCAGGS plasmid. The results of a single experiment performed with triplicate samples are shown. Error bars indicate SD. The results are representative of two separate experiments. See Supplementary Figure S3 for representative primary FRET/CFP plots.

We next asked whether coexpression of IFITM paralogues fused to SCFP and SYFP, respectively, induces FRET signals. Apart from IFITM1-3, which were all shown to display antiviral activity, we also tested IFITM5 [2,3]. As a prerequisite to these studies, all IFITM proteins were N-terminally fused to SCFP and SYFP and analyzed for expression and antiviral activity. Western blot analysis of transfected 293T cells revealed that all fusion proteins were robustly expressed, although expression of IFITM5-based fusion proteins was slightly reduced compared to that of the other IFITM proteins studied (Figure 2A). Similar results were obtained in cells engineered to stably express IFITM1-3 and the respective SCFP/SYFP fusion proteins (not shown), which were used for analysis of antiviral activity. Fusion of IFITM1-3 to fluorescent proteins was compatible with inhibition of Ebola virus glycoprotein (EBOV-GP)-driven entry, although the efficiency of inhibition by IFITM2-3-SCFP/SYFP fusion proteins was reduced compared to wild type (wt) proteins (Figure 2B). In contrast, fusion of IFITMs to fluorescent proteins largely abrogated blockade of influenza A virus hemagglutinin (FLUAV-HA)-mediated entry (Figure 2B). These results indicate different roles of the IFITM N-terminus in blockade of EBOV and FLUAV infection, although we cannot exclude that potential differences between expression of IFITM-wt and IFITM-SCFP/SYFP fusion proteins contributed to differential anti-FLUAV activity. Coexpression of IFITM1-3 and IFITM5 fused to SYFP with the identical IFITM proteins fused to SCFP resulted in robust FRET signals, and similar results were obtained for combinations of different IFITM proteins (Figure 2C, Supplementary Figure S3), indicating that these proteins have a high tendency to form homo- and heterooligomers. The only exception was observed for the reverse combination of IFITM5 fused to SCFP with IFITM1-3 fused to SYFP, which yielded low FRET signals (Figure 2C, Supplementary Figure S3). While the reasons for this phenomenon are unclear at present, it could indicate a different donor/acceptor configuration, in which reduced availability of the acceptor (in this case SYFP-IFITM5) results in reduced FRET-efficiency [43]. Apart from that, all FRET signals were more robust than those observed for SCFP/SYFP artificially targeted to membranes (Figure 1D, Supplementary Figure S2 and data not shown), suggesting signal augmentation due to real protein–protein interactions at cellular membranes. 

The amounts of plasmids transfected for FRET analysis were adjusted such that at least 90% of the cells transfected with the SYFP-SCFP positive control elicited FRET signals. Therefore, we next clarified whether potential differences in IFITM/IFITM interactions become detectable when low amounts of plasmids are transfected. To address this possibility, we analyzed FRET signals upon transfection of titrated amounts of plasmids (Figure 2D). The percentage of FRET-positive cells measured upon expression of the SYFP-SCFP positive control only slightly declined under those conditions and similar observations were made upon coexpression of IFITM3/IFITM3, although the number of positive cells declined from over 95% to roughly 80%. In contrast, the FRET signals measured upon coexpression of IFITM1 and IFTIM3 declined more steeply upon titration of plasmid, with roughly 20% of cells eliciting a FRET signal when the lowest amount of plasmid was transfected. These observations are in keeping with known differences in cellular localization of IFITM1 (mainly plasma membrane) and IFITM3 (mainly endo-/lysosomal compartments) [44,45] and suggest that subtle differences in membrane localization and/or interactions between IFITM proteins can be more readily visualized when low amounts of plasmids are transfected. Furthermore, the data indicate that IFITM3 is more likely to form homooligomers than heterooligomers with IFITM1.

Collectively, our results indicate that IFITM1–5 interact, although some differences in the extent of interaction can be visualized when low amounts of heterologous IFITM proteins are expressed.

### 2.3. F75 and F78 Are Not Required for Normal Membrane Targeting of IFITM3 and for IFITM3/IFITM3 Interactions

**Figure 3:**
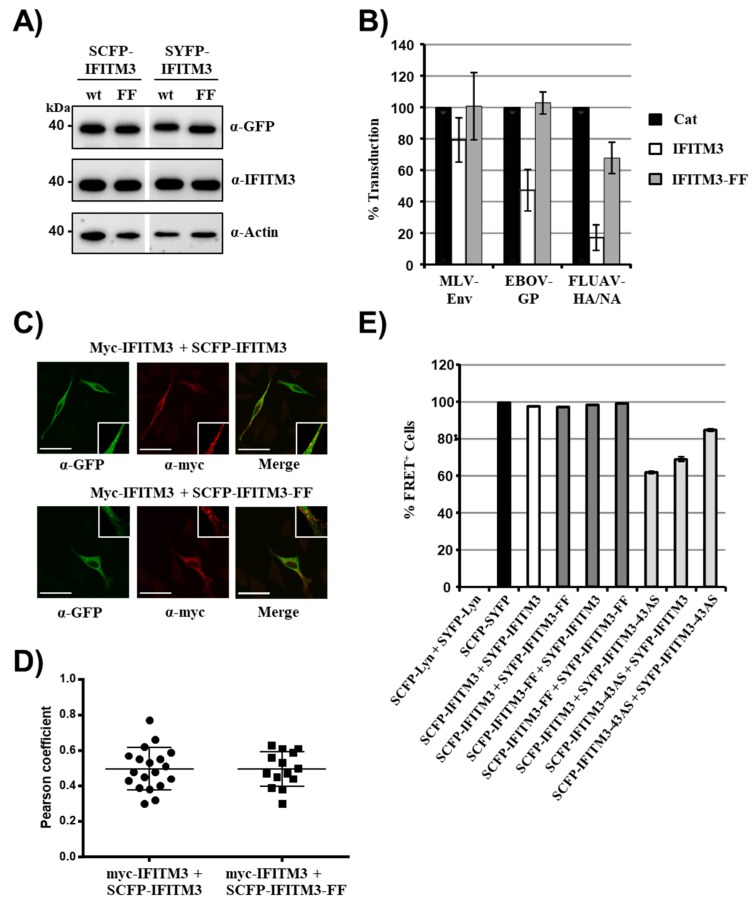
F75 and F78 in IFITM3 are dispensable for colocalization with IFITM3-wt. (**A**) 293T cells were transfected with plasmids encoding the indicated IFITM3 fusion proteins, and protein expression was analyzed by Western blot, employing anti-GFP (upper panel) and anti-IFITM3 (middle panel) antibodies for detection. Staining with anti-β-actin (lower panel) antibody served as negative control. The results of a representative blot from which irrelevant lanes were removed (white space) are shown. Similar results were obtained in two separate experiments. (**B**) 293T cells were transduced with vectors encoding IFITM3-wt, IFITM3-FF (both without antigenic tag) or Cat as control. Subsequently, the cells were transduced with infectivity normalized MLV vectors encoding firefly luciferase and pseudotyped with the indicated viral glycoproteins. At 72 h post transduction, the luciferase activities in cell lysates were determined. The average of three to four independent experiments, each carried out with triplicate samples, is shown. Error bars indicate standard error of the mean (SEM). (**C**) To analyze protein localization, HeLa cells were transfected with the indicated plasmids, stained with anti-GFP (green channel) or anti-myc (red channel) antibodies and analyzed by confocal microscopy. Scale bars indicate 50 µm. Areas of colocalization are magnified in the white squares (19.3 × 19.3 µm). (**D**) Pearson correlation coefficient analysis of the confocal images taken in panel C. (**E**) FRET analysis of 293T cells transfected with the indicated plasmid combinations at 48 h post transfection. Shown are the results of a representative experiment performed with triplicate samples, error bars indicate SD. Similar results were obtained in a separate experiment. See Supplementary Figure S4 for representative primary FRET/CFP plots.

Integrity of both F75 and F78 was previously shown to be critical for interactions between IFITM3 proteins and for antiviral activity [26]. Therefore, we asked whether mutation of both phenylalanines to alanines (mutant IFITM3-FF) was compatible with FRET in transfected 293T cells. The double mutation did not interfere with expression of IFITM3-SCFP/SYFP fusion proteins (Figure 3A) but abrogated antiviral activity (Figure 3B), as expected [26]. In contrast, the mutation of both phenylalanines did not alter colocalization of the mutant with IFITM3-wt in transfected HeLa cells (Figure 3C), as determined by analysis of the Pearson correlation coefficient (a measure for colocalization, Figure 3D). Similarly, double mutation of F75 and F78 in IFITM3 did not reduce FRET signals with coexpressed IFITM3-wt or IFITM3-FF (Figure 3E), suggesting that F75 and F78 are dispensable for appropriate localization of IFITM3 to and within membranes and for IFITM/IFITM interactions. 

The strong FRET signal observed upon coexpression of IFITM3-wt and mutant IFITM-FF raised the question whether the assay employed was sufficiently sensitive to detect potentially altered membrane localization of IFITM3 variants and/or altered protein–protein interactions. To address this concern, we first analyzed whether differences in FRET upon coexpression of IFITM3-wt/IFITM3-wt and IFITM3-wt/IFITM3-FF could be detected when the amounts of transfected plasmids were reduced as described above. However, no appreciable differences were observed (not shown). We next characterized an IFITM3 mutant, IFITM3-43AS, known to exhibit a different cellular localization as compared to IFITM3-wt [26]. In this mutant, amino acids 43–48 are changed to alanines, and this change was found to be compatible with robust expression but not with full antiviral activity [26]. Western blot analysis revealed that IFITM3-43AS fused to SCFP or SYFP was expressed with the same efficiency as the corresponding IFITM3-wt fusion proteins (not shown), as expected. Coexpression of IFITM3-43AS with IFITM3-wt reduced FRET relative to coexpression of IFITM3-wt or IFITM3-43AS proteins alone, but the signals detected remained above background (Figure 3E, Supplementary Figure S4). These results are compatible with the observation that substitution of amino acids 43–48 by alanine residues changes the cellular localization of IFTIM3 and hence the interaction with IFITM3 [26]. Moreover, they suggest that these substitutions may have only modest effects on IFITM3-43AS/IFITM3-43AS interactions. Finally, our findings suggest that the FACS-FRET assay employed in the present study can detect alterations of IFITM3/IFITM3 interactions resulting from altered membrane localization of the interactions partners.

### 2.4. F75 and F78 are Dispensable for IFITM3-IFITM3 Interactions as Determined by Co-Immunoprecipitation

**Figure 4:**
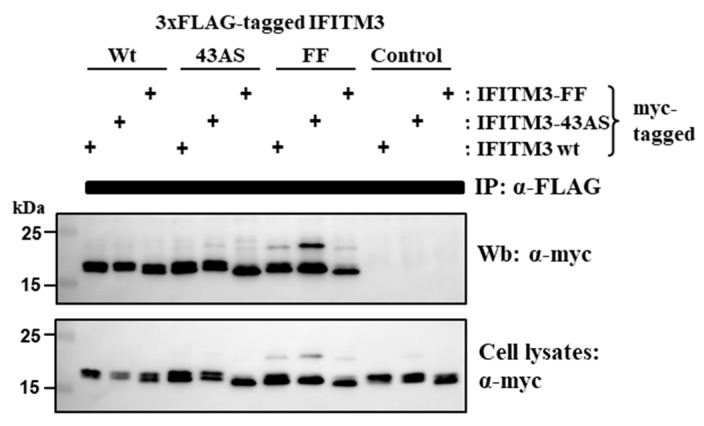
Mutation of F75 and F78 in IFITM3 does not alter homotypic interactions. 293T cells were transfected with plasmids encoding IFITM3-wt, IFITM3-FF or IFITM3-43AS tagged at the N-terminus with either 3xFLAG or myc antigenic tags. As control, an untagged IFITM3-wt-encoding plasmid was used. After 48 h, cells were lysed and proteins precipitated with murine anti-FLAG mAb (M2). Precipitates were separated by SDS-PAGE and blotted. Co-precipitated proteins were detected by murine anti-myc mAb (9E10). For comparison, cell lysates were separated and blotted in parallel and reacted with anti-myc mAb. The results are representative of three separate experiments.

Similar FRET signals were measured for the following FRET pairs, IFITM3-wt/IFITM3-wt, IFITM3-wt/IFITM3-FF and IFITM3-FF/IFITM3-FF, indicating that residues F75 and F78 are dispensable for the formation of IFITM3 homooligomers. Because F75 and F78 were previously proposed to alter the self-interaction of IFITM3 [26], we sought to corroborate this result by an independent experimental approach, coimmunoprecipitation (Figure 4). For this, IFITM3-wt and IFITM3-FF were equipped with a triple FLAG antigenic tag, controlled for comparable expression (not shown) and coexpressed with the same proteins harboring a myc tag. Mutant IFITM-43AS was included for comparison. Expression of untagged IFITM3-wt instead of IFTIM3-wt with FLAG tag served as negative control. Immunoprecipitation with anti-FLAG and subsequent Western blot with anti-myc antibody revealed that IFITM3-wt proteins interacted and that this interaction was not perturbed by substitution of amino acids 43–48 with alanine residues. Again, this finding was in-line with the FRET results, showing that FRET signals of IFITM3 with IFITM3-43AS are reduced but not fully disrupted (Figure 4), indicating that both proteins can still interact in membrane microdomains. F75 and F78 were also dispensable for interactions with IFITM3-wt and IFITM-FF, which contrasts with published data [26] but is in keeping with the FRET analysis discussed above. In summary, our results indicate that F75 and F78 are neither required for adequate membrane targeting of IFITM3 nor for IFITM3/IFITM3 interactions.

## 3. Discussion

IFITM3 poses an important innate barrier against infection by several enveloped viruses [1] and a non-enveloped virus [46]. Therefore, it is important to understand how IFITM3 counters virus infection. Published work indicates that IFITM3/IFITM3 interactions are required for antiviral activity and depend on F75 and F78 [26]. Here, we employed a previously described FACS-FRET assay [30] for analysis of IFTIM/IFITM interactions. We provide evidence for interactions between IFITM1-3 and 5, and we show that IFITM3-wt and IFITM3 with mutated F75 and F78 induce robust FRET signals and physically interact, indicating that F75 and F78 are dispensable for adequate membrane localization and IFITM3-IFITM3 interactions.

We first asked whether FRET signals in our assay depend on protein–protein interactions or whether targeting of two non-interacting proteins to membranes can be sufficient for FRET. The latter was the case: SCFP and SYFP did not elicit FRET signals when expressed in the cytosol while FRET was observed when both proteins were targeted to the plasma membrane. This observation raised the question whether membrane targeting alone was sufficient for FRET or whether targeting to membrane subdomains was required. SYFP and SCFP equipped with a geranylgeranylation-dependent membrane targeting signal did not elicit FRET, despite robust expression and membrane localization. These findings suggest that membrane localization alone was not sufficient for FRET in the assay employed. Finally, it should be noted that FRET signals measured upon membrane targeting of SCFP and SYFP were modest while robust signals were measured for SCFP and SYFP fused to IFITM3-wt, a membrane targeted protein known to form homotypic interactions [26,47]. Collectively, FRET signals solely due to localization of proteins in the same membrane subdomain were detectable but low while high signals were measured upon protein–protein interactions of membrane proteins. IFITM proteins inhibit host cell entry of several enveloped viruses and are believed to exert their antiviral activity by altering membrane fluidity and/or curvature [22,23]. This might require the formation of IFITM multimers due to IFITM-IFITM interactions, and a previous study indeed showed homotypic interactions between IFITM proteins [26]. The present results are in agreement with these findings and provide evidence that also IFITM5 proteins may self-interact and interact with other IFITM paralogues. Why the FRET signals measured upon IFITM5 coexpression with IFITM1–3 were asymmetric, i.e., dependent on whether IFITM5 was donor or acceptor, is at present unclear, and differences in donor/acceptor configuration might be responsible, as stated above. Alternatively, the results may point towards differences in the heterotypic interactions between IFITM1–3 and IFITM5/IFITM1–3.

Previous work by John and colleagues found that amino acid residues in IM1 and a conserved intracellular loop (CIL), which jointly form the CD225 domain of IFITM3, are important for antiviral activity [26]. In particular, the double mutation of F75 and F78 strongly reduced antiviral activity and this phenotype was associated with lack of IFITM3/IFITM3 interactions [26]. Our results confirm the loss of antiviral activity since F75 and F78 were required for blockade of both EBOV-GP- and FLUAV-HA-mediated entry. The lack of antiviral activity of IFITM3 with mutated F75 and F78 might stem from altered membrane localization of the mutant relative to the wt protein or could be due to abrogated IFITM3/IFITM3 interactions, as previously suggested [26]. The present study suggests that the membrane localization of IFITM3-wt and IFITM3-FF was not substantially different, since FRET signals in cells coexpressing IFITM3-wt/IFITM3-wt or IFITM3-wt/IFITM3-FF mutant were comparable. Moreover, coimmunoprecipitation showed that mutation of phenylalanine 75 and 78 was compatible with robust interactions with IFITM3-wt and IFITM3-FF. These results are not in keeping with previously published data [26], potentially due to differences in expression levels and conditions used for coimmunoprecipitation. 

Collectively, we report an experimental system to measure interactions between IFITM proteins and provide evidence that residues F75 and F78 are required for antiviral activity but not for normal membrane localization and IFITM3/IFITM3 interactions.

## 4. Materials and Methods 

### 4.1. Cell Culture

HEK 293T cells (DSMZ ACC 635) and HeLa cells (ATCC CCL-2) were maintained in Dulbecco’s Modified Eagle Medium (DMEM), while COS7 cells (ATCC CRL-1651) were cultivated in Minimal Essential Medium (MEM). All cell culture media were supplemented with 10% fetal calf serum, 2 mM L-glutamine, 100 U/mL penicillin and 100 μg/mL streptomycin. The cell lines were obtained from collaborators, and the identity of the human cell lines was verified by STR typing [48].

### 4.2. Plasmid Construction 

Plasmids encoding the glycoproteins of vesicular stomatitis virus (VSV-G), murine leukemia virus (MLV Env), Ebola virus (EBOV-GP) and FLUAV strain WSN (FLUAV-HA/NA) as well as plasmids MLV gag-pol and MLV luc have been described before [49,50]. Human IFITM5 was assembled from individual exons by splice-overlap PCR using primers hIFITM5-5Not, hIFITM5-i3, hIFITM5-i5, hIFITM5-3Eco and cloned into plasmid pQCXIP-mcs via NotI and EcoRI. Plasmids pSYFP2-C1 and pSCFP3A-NES were previously described [35]. Plasmids pSCFP3A-C1, pSCFP3A-N1 and pSYFP2-N1 were generated by exchanging AgeI/BsrGI fragments encoding fluorescent protein sequences in pEYFP-C1 or pEYFP-N1 (both from Clontech, Mountain View, CA, USA) with the respective fragments of pSYFP2-C1 and pSCFP3A-NES. To generate a FRET positive control, a SmaI and XbaI SCFP-Fragment from pSCFP-N1 was ligated to pSYFP2-C1 digested with Ecl136II and XbaI to give rise to pSYFP-SCFP, which encodes a SYFP-SCFP fusion protein. 

In order to target SYFP and SCFP to the membrane, fusion proteins with the membrane localization of signals of neuromodulin, Lyn and RhoA were generated. For fusing neuromodulin membrane targeting signal to the N-terminus of SCFP and SYFP, the phosphorylated oligonucleotides Mem-AccAge-for and Mem-AccAge-rev were ligated into pSCFP3A-N1 or pSYFP2-N1 digested with Acc65I and AgeI, resulting in plasmids pSCFP3A-NM-Mem and pSYFP2-NM-Mem, respectively. For fusion of the Lyn membrane targeting signal to the N-terminus of the fluorescent proteins, SCFP3A or SYFP2 were amplified using primers Myr-Pal-GFP-5E and SV40pAseq, digested with EcoRI and NotI and ligated into plasmid pEYFP-N1, giving rise to plasmids pSCFP3A-N-MyrPal and pSYFP2-N-MyrPal, respectively. Finally, the membrane targeting signal of RhoA was fused to the C-terminus of the fluorescent proteins via PCR-amplification of SCFP3A and SYFP2 with primers HCMVep-seq and ScyFP-Gerc and cloning of the AgeI and EcoRI-digested product into pSCFP3A-C1, resulting in plasmids pSCFP3A-C-Ger and pSYFP2-C-Ger, respectively. 

Plasmids encoding IFITM1-3 and 5 fused to SCFP3A or SYFP2 were generated by subcloning the coding sequences as NotI/EcoRI fragments from pCAGGS-IFITM1-3 [51] into pSCFP3A-C1 or pSYFP2-C1 [15]. For generation of retroviral vectors encoding SxFP-IFITM fusion proteins, the SCFP3A-IFITM1 or SYFP2-IFITM1 coding sequences were amplified with primers EIF3-5Esp and SV40pAseq digested with Esp3I and EcoRI and cloned into pQCXIP-mcs cut with NotI and EcoRI. Finally, NotI/EcoRI fragments encoding IFITM2 and 3 sequences were used to replace IFITM1 to generate pQCXIP encoding the respective SxFP-IFITM2 and 3 fusion proteins [15]. Plasmid encoding IFITM3 mutant 43AS was obtained from Abraham Brass (pQCXIP-IFITM3 43-48 AS). The IFITM3-FF mutant was generated by overlap-extension PCR using pCAGGS-IFITM3 [51] as template and primer pairs IFITM3-5N/mut-IFITM3-F75AF78A-rev and mut-IFITM3-F75AF78A-for/pCAGGS-3′ to amplify parts of the IFITM3 gene. The PCR product was cloned into pQCXIP using NotI and EcoRI. Subsequently, IFITM3-43AS and IFITM3-FF were subcloned as NotI/EcoRI fragments into pSCFP3A-IFITM1 or pSYFP2-IFITM1 thus replacing the IFITM1 genes. To generate pcDNA3-myc1-IFITM3, the IFITM3 gene was subcloned as Acc65I and EcoRI fragment from pCAGGS-IFITM3 into pcDNA3-myc1, which contains start codon and myc-epitope coding sequences inserted into HindIII and Acc65I sites of pcDNA3 (Invitrogen, Carlsbad, CA, USA). Flag tagged IFITM3 was generated by splice overlap PCR using HCMVep-seq and FLAG-IFrev primers and pCMV-3xFLAG-SNX5 [52] template as well as template pCAGGS-IFITM3 and primers FLAG-IFfor and pCAGGS-3′ and cloned into pcDNA3 via HindIII and EcoRI. Mutant genes IFITM3-43AS and IFITM3-FF were inserted into pcDNA3-myc1 and pcDNA3-3xFLAG as Acc65I and BamHI sites fragments from the respective pQCXIP-plasmids. The integrity of all constructs was confirmed by automated sequencing.

### 4.3. Oligonucleotides

Oligonucleotides were purchased from Sigma-Aldrich (St. Louis, MO, USA): 

hIFITM5-5Not 5′-CCGCGGCCGCACCATGGACACGGCGTATCCCCGCGAG-3′, hIFITM5-i3 5′-ACCACCTTCTGATCTCGGGCCTTGATGGAGTAGGCCAGCG-3′, hIFITM5-i5 5′-CGCTGGCCTACTCCATCAAGGCCCGAGATCAGAAGGTGGT-3′, hIFITM5-3Eco 5′-CGAATTCTCAGTCATAGTCCGCGTCATCAAAC-3′, Mem-AccAge-for 5′- GTACCACCATGCTGTGCTGTATGAGAAGAACCAAACAGGTTGAAAAGAATGATGAGGACCAAAAGATA-3′, Mem-AccAge-rev 5′- CCGGTATCTTTTGGTCCTCATCATTCTTTTCAACCTGTTTGGTTCTTCTCATACAGCACAGCATGGTG-3′, Myr-Pal-GFP-5E 5′- CGAATTCACCATGGGCTGCATCAAGAGCAAGCGCAAGGACAACCTGAACGACGACGAGCCACCGGTCGCCACCATGGTG-3′, SV40pAseq 5′-GAAATTTGTGATGCTATTGC-3, HCMVep-seq 5-GCAAATGGGCGGTAGGCGTG-3′, ScyFP-Ger 5′- GGAATTCTCACAAGACAAGGCACCCAGAAGATCTGAGTCCGGACTTG-3′, EIF3-5Esp 5′-GACGTCTCAGGCCACGCGTGCTAGCACCATGGTGAGCAAGGG-3′, SV40pAseq 5′-GAAATTTGTGATGCTATTGC-3′, IFITM3-5N 5′- GCGCGGCCGCACCATGAATCACACTGTCCAAACC-3′, mut-IFITM3-F75AF78A-rev 5′- GACTTCACGGAGTAGGCAGCTGCTATGGCGCCCAGGCAGCAGGG-3′, mut-IFITM3-F75AF78A-for 5′-CCCTGCTGCCTGGGCGCCATAGCAGCTGCCTACTCCGTGAAGTC-3′, pCAGGS-3′ 5′- CAGAAGTCAGATGCTCAAGGG-3′

### 4.4. Antibodies

Rabbit anti-GFP polyclonal serum (BioVision, Milpitas, CA, USA) was used for immunoblot at 1:500 dilution. IFITM proteins were detected with a mouse monoclonal anti-IFITM1 antibody (Proteintech Group, Manchester, UK; diluted 1:500) and a rabbit anti-IFITM2 antiserum, which is cross-reactive with IFITM3 (Proteintech, Chicago, IL, USA; diluted 1:1000).

### 4.5. Immunofluorescence 

Cos-7 or HeLa cells were seeded into 24-well plates containing coverslips (12 mm diameter) and transfected the next day with 1 µg of each plasmid by calcium phosphate precipitation. Two days later transfected cells were fixed with 4% paraformaldehyde for 10 min at room temperature (RT). After four washes with PBS, cells were permeabilized with 0.2% Triton X-100 in PBS for 2 min at RT followed by three wash steps with PBS. Cells were then incubated for 30 min at 37 °C in cell culture supernatant from anti-myc hybridoma cells (9E10) and rabbit anti-GFP (1:500) which were used as primary antibodies. After three wash steps incubation with secondary antibodies anti-rabbit Alexafluor488 (1:1000, Thermo Fisher, Waltham, MA, USA), anti-mouse Alexafluor546 (1:1000, Thermo Fisher) followed for 30 min at 37 °C. After three final washes in PBS, cells were mounted in Mowiol/DABCO. Images were taken on a confocal microscope (Zeiss, Oberkochen, Germany, LSM 510 Meta equipped with 405 and 488 nm laser sources or Zeiss LSM 5 Pascal equipped with 488 and 543 nm laser sources) using 63x/1.40 Oil Plan-Apochromat objectives on both microscopes. Calculation of the Pearson correlation coefficient was performed using ImageJ software with Just Another Colocalization Plugin [53].

### 4.6. Immunoblot

For analysis of protein expression by immunoblot protein lysates were separated by SDS-PAGE and subsequently transferred onto nitrocellulose membranes (Whatman Protan BA 83 0.2 µm, GE Healthcare, Berlin, Germany). Membranes were blocked in PBS with 0.1 % Triton X-100 (PBS-T) and 5% milk powder for at least one hour. Membranes were incubated with primary antibody diluted in PBS-T in 50 mL Falcon tubes for one hour on a laboratory roller and subsequently washed three times for 5 min in PBS-T. Bound antibodies were detected using the ECL Prime Western Blotting Detection Reagents (GE Healthcare, Berlin, Germany) according to the protocols of the manufacturer. Signals were visualized with the ChemoCam imaging system equipped with the ChemoStarProfessional software (Intas, Göttingen, Germany).

### 4.7. Production of Retroviral Vectors and Transduction Experiments

For production of retroviral vectors pseudotyped with different viral glycoproteins, HEK 293T cells were seeded in T25 flasks at 60%–70% confluency. The next day, cells were cotransfected with plasmids MLV gag-pol (3 µg) and MLV luc (6 µg) and a plasmid encoding a viral glycoprotein (3 µg), employing the calcium-phosphate-precipitation method. For production of vectors encoding IFITM proteins, plasmids MLV gag-pol (3 µg), VSV-G (3 µg) and a plasmid encoding IFITM or a control gene (CAT, 6 µg) were cotransfected. The cell culture medium was exchanged at 6–8 h after transfection and culture supernatants were harvested after 48 h. The supernatants were cleared by filtration through a 0.45 µm filter, aliquoted and stored at –80 °C. In order to analyse inhibition of viral entry by IFITM proteins, HEK 293T cells were seeded in 96-well plates at 10,000 cells/well. The next day, cells were transduced with vectors encoding IFITMs or Cat control by spinoculation (centrifugation at 4000× *g* for 30 min) followed by 48 h incubation. Vector-containing supernatants were then replaced by 50 µL fresh culture medium followed by transduction with 50 µL of supernatants containing particles pseudotyped with different envelope proteins. After incubation for 8 h, culture supernatants were replaced by 150 µL fresh culture medium. After 72 h, cells were lysed, and luciferase activity in cell lysates was quantified.

### 4.8. Coimmunoprecipitation

HEK 293T cells were seeded in 6-well plates at 250,000 cells/well and transfected by calcium-phosphate transfection on the next day with each 2 µg of plasmids encoding myc- or Flag-tagged IFITM proteins. Cells were harvested after two days and lysed in 500 µL CoIP-buffer (50 mM Tris pH 7.4, 150mM NaCl, 0.5% CHAPSO). For precipitation, 1 µL (per sample) mouse-anti-FLAG (M2) (Agilent, Santa Clara, CA, USA) was reacted for 1 h at 4 °C with Protein A/G PLUS-Agarose (Santa Cruz Biotechnology, Heidelberg, Germany) equilibrated in CoIP-buffer and subsequently mixed with cell lysate samples. The samples were incubated on a rotator at room temperature for 30 min and then centrifuged to collect the precipitate. After five washing steps in CoIP-buffer, the precipitates were separated by SDS-PAGE and subjected to immunoblot analysis. Co-immunoprecipitated proteins were detected with mouse anti-myc (9E10) mAb and Clean-Blot Detection Reagent (Thermo-Fisher, Waltham, MA, USA). In parallel, unprecipitated cell lysates were subjected to SDS-PAGE and immunoblot.

### 4.9. Flow-Cytometry and FRET

HEK 293T cells were seeded in 6-well plates at 250,000 cells/well and transfected by calcium-phosphate transfection on the next day, usually with 1 µg of each plasmid encoding the FRET-partners. Cells were harvested after 1.5 days in ice-cold PBS and kept on ice until analyzed. Flow-cytometry FRET measurements [30] were performed using a LSR II (BD Biosciences, San Jose, CA, USA) equipped with 405 nm and 488 nm lasers. Cells were gated to measure only single cells but exclude doublets or larger aggregates. To measure SCFP and FRET signals, cells were excited with the 405 nm laser and fluorescence was collected in the SCFP channel with a standard 450/40 filter, while the FRET signal was measured with a 530/30 filter. To measure the SYFP signal, cells were excited with the 488 nm laser and emission was also collected with a 530/30 filter. To determine FRET-signals SCFP and SYFP, channels were first compensated using single positive cells, expressing either SCFP-Ger or SYFP-Ger. In a FRET/SYFP, plot cells were gated to just exclude signals arising from excitation of SYFP at 405 nm due to high expression levels. Then, double positive cells were gated in a SCFP/SYFP plot. Finally, in a FRET/SCFP plot, the gate was adjusted to just exclude signals from the positive control (cells expressing SCFP-SYFP fusion protein), thus applying stringent criteria for detection of strong interactions. The gating strategy is shown in Supplementary Figure S1.

## Supplementary Materials

Supplementary materials can be found at https://www.mdpi.com/1422-0067/20/16/3859/s1.

## Abbreviations

EBOV	Ebola virus
eCFP	enhanced cyan fluorescent protein
eYFP	enhanced yellow fluorescent protein
FLUAV	Influenza A virus
FRET	Fluorescence resonance energy transfer
IFITM	Interferon-induced transmembrane protein
MLV	Murine leukemia virus
SCFP	super cyan fluorescent protein
SYFP	super yellow fluorescent protein
wt	wildtype

## References

[1] Shi G., Schwartz O., Compton A.A. (2017). More than meets the I: The diverse antiviral and cellular functions of interferon-induced transmembrane proteins. Retrovirology.

[2] Brass A.L., Huang I.C., Benita Y., John S.P., Krishnan M.N., Feeley E.M., Ryan B.J., Weyer J.L., van der Weyden L., Fikrig E. (2009). The IFITM proteins mediate cellular resistance to influenza A H1N1 virus, West Nile virus, and dengue virus. Cell.

[3] Huang I.C., Bailey C.C., Weyer J.L., Radoshitzky S.R., Becker M.M., Chiang J.J., Brass A.L., Ahmed A.A., Chi X., Dong L. (2011). Distinct patterns of IFITM-mediated restriction of filoviruses, SARS coronavirus, and influenza A virus. PLoS Pathog..

[4] Feeley E.M., Sims J.S., John S.P., Chin C.R., Pertel T., Chen L.M., Gaiha G.D., Ryan B.J., Donis R.O., Elledge S.J. (2011). IFITM3 inhibits influenza A virus infection by preventing cytosolic entry. PLoS Pathog..

[5] Everitt A.R., Clare S., Pertel T., John S.P., Wash R.S., Smith S.E., Chin C.R., Feeley E.M., Sims J.S., Adams D.J. (2012). IFITM3 restricts the morbidity and mortality associated with influenza. Nature.

[6] Kim Y.C., Jeong B.H. (2017). No Correlation of the Disease Severity of Influenza A Virus Infection with the rs12252 Polymorphism of the Interferon-Induced Transmembrane Protein 3 Gene. Intervirology.

[7] Mills T.C., Rautanen A., Elliott K.S., Parks T., Naranbhai V., Ieven M.M., Butler C.C., Little P., Verheij T., Garrard C.S. (2014). IFITM3 and susceptibility to respiratory viral infections in the community. J. Infect. Dis..

[8] Bailey C.C., Huang I.C., Kam C., Farzan M. (2012). Ifitm3 limits the severity of acute influenza in mice. PLoS Pathog..

[9] Compton A.A., Bruel T., Porrot F., Mallet A., Sachse M., Euvrard M., Liang C., Casartelli N., Schwartz O. (2014). IFITM proteins incorporated into HIV-1 virions impair viral fusion and spread. Cell Host Microbe.

[10] Foster T.L., Wilson H., Iyer S.S., Coss K., Doores K., Smith S., Kellam P., Finzi A., Borrow P., Hahn B.H. (2016). Resistance of Transmitted Founder HIV-1 to IFITM-Mediated Restriction. Cell Host Microbe.

[11] Roesch F., OhAinle M., Emerman M. (2018). A CRISPR screen for factors regulating SAMHD1 degradation identifies IFITMs as potent inhibitors of lentiviral particle delivery. Retrovirology.

[12] Tartour K., Appourchaux R., Gaillard J., Nguyen X.N., Durand S., Turpin J., Beaumont E., Roch E., Berger G., Mahieux R. (2014). IFITM proteins are incorporated onto HIV-1 virion particles and negatively imprint their infectivity. Retrovirology.

[13] Poddar S., Hyde J.L., Gorman M.J., Farzan M., Diamond M.S. (2016). The Interferon-Stimulated Gene IFITM3 Restricts Infection and Pathogenesis of Arthritogenic and Encephalitic Alphaviruses. J. Virol..

[14] Weston S., Czieso S., White I.J., Smith S.E., Wash R.S., Diaz-Soria C., Kellam P., Marsh M. (2016). Alphavirus Restriction by IFITM Proteins. Traffic.

[15] Wrensch F., Karsten C.B., Gnirss K., Hoffmann M., Lu K., Takada A., Winkler M., Simmons G., Pöhlmann S. (2015). Interferon-Induced Transmembrane Protein-Mediated Inhibition of Host Cell Entry of Ebolaviruses. J. Infect. Dis..

[16] Chan Y.K., Huang I.C., Farzan M. (2012). IFITM proteins restrict antibody-dependent enhancement of dengue virus infection. PLoS ONE.

[17] Savidis G., Perreira J.M., Portmann J.M., Meraner P., Guo Z., Green S., Brass A.L. (2016). The IFITMs Inhibit Zika Virus Replication. Cell Rep..

[18] Gorman M.J., Poddar S., Farzan M., Diamond M.S. (2016). The Interferon-Stimulated Gene Ifitm3 Restricts West Nile Virus Infection and Pathogenesis. J. Virol..

[19] Xie M., Xuan B., Shan J., Pan D., Sun Y., Shan Z., Zhang J., Yu D., Li B., Qian Z. (2015). Human cytomegalovirus exploits interferon-induced transmembrane proteins to facilitate morphogenesis of the virion assembly compartment. J. Virol..

[20] Zhao X., Guo F., Liu F., Cuconati A., Chang J., Block T.M., Guo J.T. (2014). Interferon induction of IFITM proteins promotes infection by human coronavirus OC43. Proc. Natl. Acad. Sci. USA.

[21] Zhao X., Sehgal M., Hou Z., Cheng J., Shu S., Wu S., Guo F., Le Marchand S.J., Lin H., Chang J. (2018). Identification of Residues Controlling Restriction versus Enhancing Activities of IFITM Proteins on Entry of Human Coronaviruses. J. Virol..

[22] Desai T.M., Marin M., Chin C.R., Savidis G., Brass A.L., Melikyan G.B. (2014). IFITM3 restricts influenza A virus entry by blocking the formation of fusion pores following virus-endosome hemifusion. PLoS Pathog..

[23] Li K., Markosyan R.M., Zheng Y.M., Golfetto O., Bungart B., Li M., Ding S., He Y., Liang C., Lee J.C. (2013). IFITM proteins restrict viral membrane hemifusion. PLoS Pathog..

[24] Amini-Bavil-Olyaee S., Choi Y.J., Lee J.H., Shi M., Huang I.C., Farzan M., Jung J.U. (2013). The antiviral effector IFITM3 disrupts intracellular cholesterol homeostasis to block viral entry. Cell Host Microbe.

[25] Lin T.Y., Chin C.R., Everitt A.R., Clare S., Perreira J.M., Savidis G., Aker A.M., John S.P., Sarlah D., Carreira E.M. (2013). Amphotericin B increases influenza A virus infection by preventing IFITM3-mediated restriction. Cell Rep..

[26] John S.P., Chin C.R., Perreira J.M., Feeley E.M., Aker A.M., Savidis G., Smith S.E., Elia A.E., Everitt A.R., Vora M. (2013). The CD225 domain of IFITM3 is required for both IFITM protein association and inhibition of influenza A virus and dengue virus replication. J. Virol..

[27] Yount J.S., Karssemeijer R.A., Hang H.C. (2012). S-palmitoylation and ubiquitination differentially regulate interferon-induced transmembrane protein 3 (IFITM3)-mediated resistance to influenza virus. J. Biol. Chem..

[28] Yount J.S., Moltedo B., Yang Y.Y., Charron G., Moran T.M., Lopez C.B., Hang H.C. (2010). Palmitoylome profiling reveals S-palmitoylation-dependent antiviral activity of IFITM3. Nat. Chem. Biol..

[29] McMichael T.M., Zhang L., Chemudupati M., Hach J.C., Kenney A.D., Hang H.C., Yount J.S. (2017). The palmitoyltransferase ZDHHC20 enhances interferon-induced transmembrane protein 3 (IFITM3) palmitoylation and antiviral activity. J. Biol. Chem..

[30] Banning C., Votteler J., Hoffmann D., Koppensteiner H., Warmer M., Reimer R., Kirchhoff F., Schubert U., Hauber J., Schindler M. (2010). A flow cytometry-based FRET assay to identify and analyse protein-protein interactions in living cells. PLoS ONE.

[31] Hagen N., Bayer K., Rosch K., Schindler M. (2014). The intraviral protein interaction network of hepatitis C virus. Mol. Cell. Proteom..

[32] Gondim M.V., Wiltzer-Bach L., Maurer B., Banning C., Arganaraz E., Schindler M. (2015). AP-2 Is the Crucial Clathrin Adaptor Protein for CD4 Downmodulation by HIV-1 Nef in Infected Primary CD4+ T Cells. J. Virol..

[33] Suffner S., Gerstenberg N., Patra M., Ruibal P., Orabi A., Schindler M., Bruss V. (2018). Domains of the Hepatitis B Virus Small Surface Protein S Mediating Oligomerization. J. Virol..

[34] Lam A.J., St-Pierre F., Gong Y., Marshall J.D., Cranfill P.J., Baird M.A., McKeown M.R., Wiedenmann J., Davidson M.W., Schnitzer M.J. (2012). Improving FRET dynamic range with bright green and red fluorescent proteins. Nat. Methods.

[35] Kremers G.J., Goedhart J., van Munster E.B., Gadella T.W. (2006). Cyan and yellow super fluorescent proteins with improved brightness, protein folding, and FRET Forster radius. Biochemistry.

[36] Skene J.H., Virag I. (1989). Posttranslational membrane attachment and dynamic fatty acylation of a neuronal growth cone protein, GAP-43. J. Cell Biol..

[37] Sudo Y., Valenzuela D., Beck-Sickinger A.G., Fishman M.C., Strittmatter S.M. (1992). Palmitoylation alters protein activity: Blockade of G(o) stimulation by GAP-43. EMBO J..

[38] Kovarova M., Tolar P., Arudchandran R., Draberova L., Rivera J., Draber P. (2001). Structure-function analysis of Lyn kinase association with lipid rafts and initiation of early signaling events after Fcepsilon receptor I aggregation. Mol. Cell. Biol..

[39] Adamson P., Paterson H.F., Hall A. (1992). Intracellular localization of the P21rho proteins. J. Cell Biol..

[40] Hori Y., Kikuchi A., Isomura M., Katayama M., Miura Y., Fujioka H., Kaibuchi K., Takai Y. (1991). Post-translational modifications of the C-terminal region of the rho protein are important for its interaction with membranes and the stimulatory and inhibitory GDP/GTP exchange proteins. Oncogene.

[41] Seibel N.M., Eljouni J., Nalaskowski M.M., Hampe W. (2007). Nuclear localization of enhanced green fluorescent protein homomultimers. Anal. Biochem..

[42] De Almeida R.F., Loura L.M., Fedorov A., Prieto M. (2005). Lipid rafts have different sizes depending on membrane composition: A time-resolved fluorescence resonance energy transfer study. J. Mol. Biol..

[43] Ben-Johny M., Yue D.N., Yue D.T. (2016). Detecting stoichiometry of macromolecular complexes in live cells using FRET. Nat. Commun..

[44] Lu J., Pan Q., Rong L., He W., Liu S.L., Liang C. (2011). The IFITM proteins inhibit HIV-1 infection. J. Virol..

[45] Narayana S.K., Helbig K.J., McCartney E.M., Eyre N.S., Bull R.A., Eltahla A., Lloyd A.R., Beard M.R. (2015). The Interferon-induced Transmembrane Proteins, IFITM1, IFITM2, and IFITM3 Inhibit Hepatitis C Virus Entry. J. Biol. Chem..

[46] Anafu A.A., Bowen C.H., Chin C.R., Brass A.L., Holm G.H. (2013). Interferon-inducible transmembrane protein 3 (IFITM3) restricts reovirus cell entry. J. Biol. Chem..

[47] Bailey C.C., Zhong G., Huang I.C., Farzan M. (2014). IFITM-Family Proteins: The Cell’s First Line of Antiviral Defense. Annu. Rev. Virol..

[48] Dirks W.G., Drexler H.G. (2013). STR DNA typing of human cell lines: Detection of intra- and interspecies cross-contamination. Methods Mol. Biol..

[49] Bartosch B., Dubuisson J., Cosset F.L. (2003). Infectious hepatitis C virus pseudo-particles containing functional E1-E2 envelope protein complexes. J. Exp. Med..

[50] Wrensch F., Hoffmann M., Gartner S., Nehlmeier I., Winkler M., Pöhlmann S. (2017). Virion Background and Efficiency of Virion Incorporation Determine Susceptibility of Simian Immunodeficiency Virus Env-Driven Viral Entry to Inhibition by IFITM Proteins. J. Virol..

[51] Bertram S., Dijkman R., Habjan M., Heurich A., Gierer S., Glowacka I., Welsch K., Winkler M., Schneider H., Hofmann-Winkler H. (2013). TMPRSS2 activates the human coronavirus 229E for cathepsin-independent host cell entry and is expressed in viral target cells in the respiratory epithelium. J. Virol..

[52] Teasdale R.D., Loci D., Houghton F., Karlsson L., Gleeson P.A. (2001). A large family of endosome-localized proteins related to sorting nexin 1. Biochem. J..

[53] Bolte S., Cordelieres F.P. (2006). A guided tour into subcellular colocalization analysis in light microscopy. J. Microsc.

