# Supplementary material for: Analysis of IFITM-IFITM Interactions by a Flow Cytometry-Based FRET Assay
Source: Int J Mol Sci. 2019 Aug 8;20(16):3859. doi: 10.3390/ijms20163859 (PMC6719045; doi:10.3390/ijms20163859)
Supplement: Supplementary file 1 [file ijms-20-03859-s001.zip › ijms-554012-supplementary.pdf]

Suppl. Fig. 1.

Winkler *et al.*

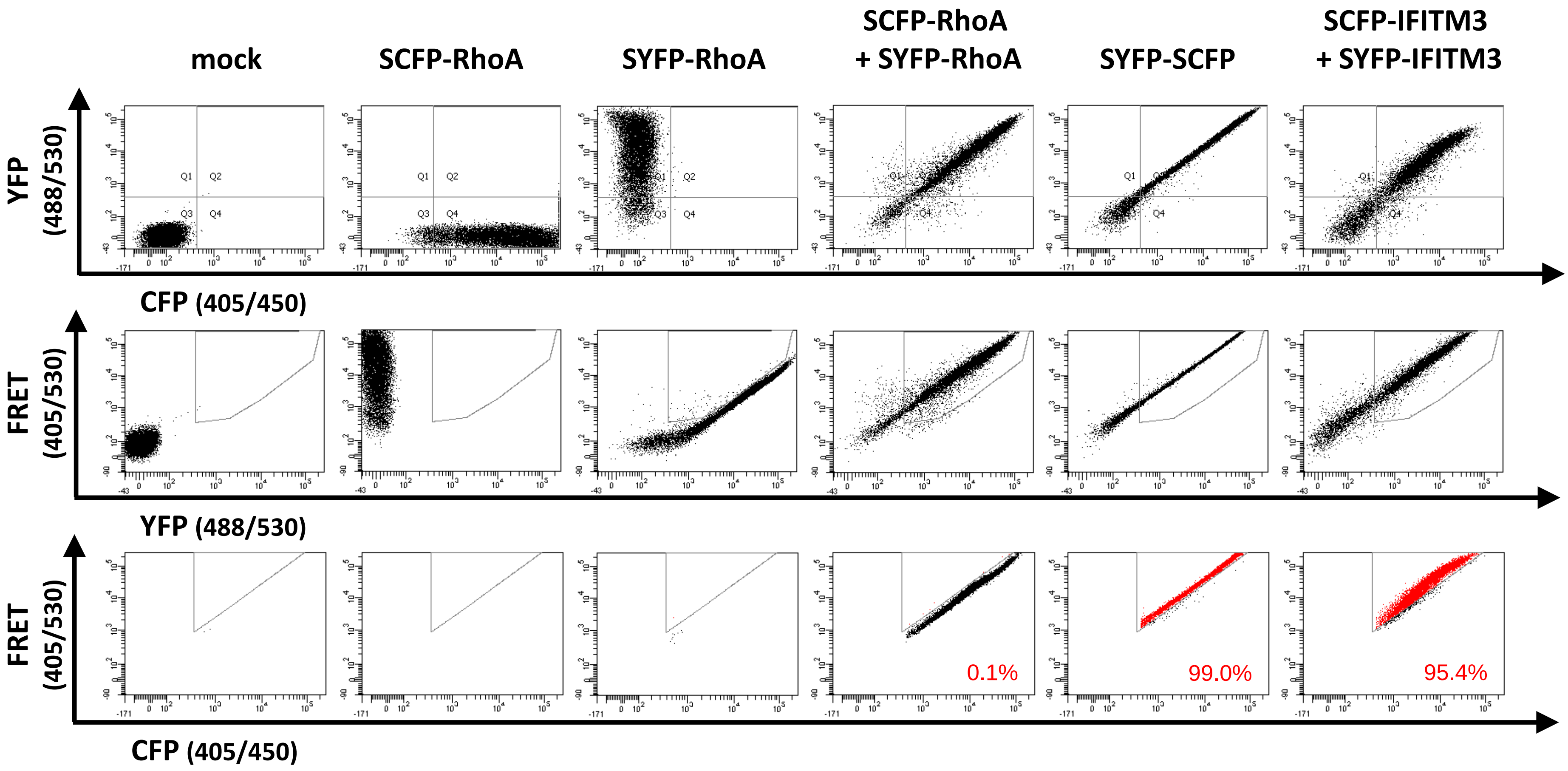

Suppl. Figure 1. Gating strategy used for FACS-FRET measurements. 293T cells were transfected with plasmids encoding the proteins indicated on top. Living cells were analyzed in a LSR II flow cytometer. First, in a plot YFP vs. CFP a double positive cell population was gated (top panels). In a second step, cells false positive for FRET signals due to high SYFP signals were excluded in a FRET vs. YFP plot (middle panels). Finally, a FRET vs. CFP plot was used to define a gate (bottom panel) which excludes signals arising from SCFP-RhoA and SYFP-RhoA coexpression (FRET negative) and includes signals arising from SYFP-SCFP fusion proteins (FRET positive). FRET positive cells are colored red and the percentage of FRET-positive cells is given for negative and positive controls and a SCFP-IFITM3 + SYFP-IFITM3 sample.

Suppl. Fig. 2.

Winkler *et al.*

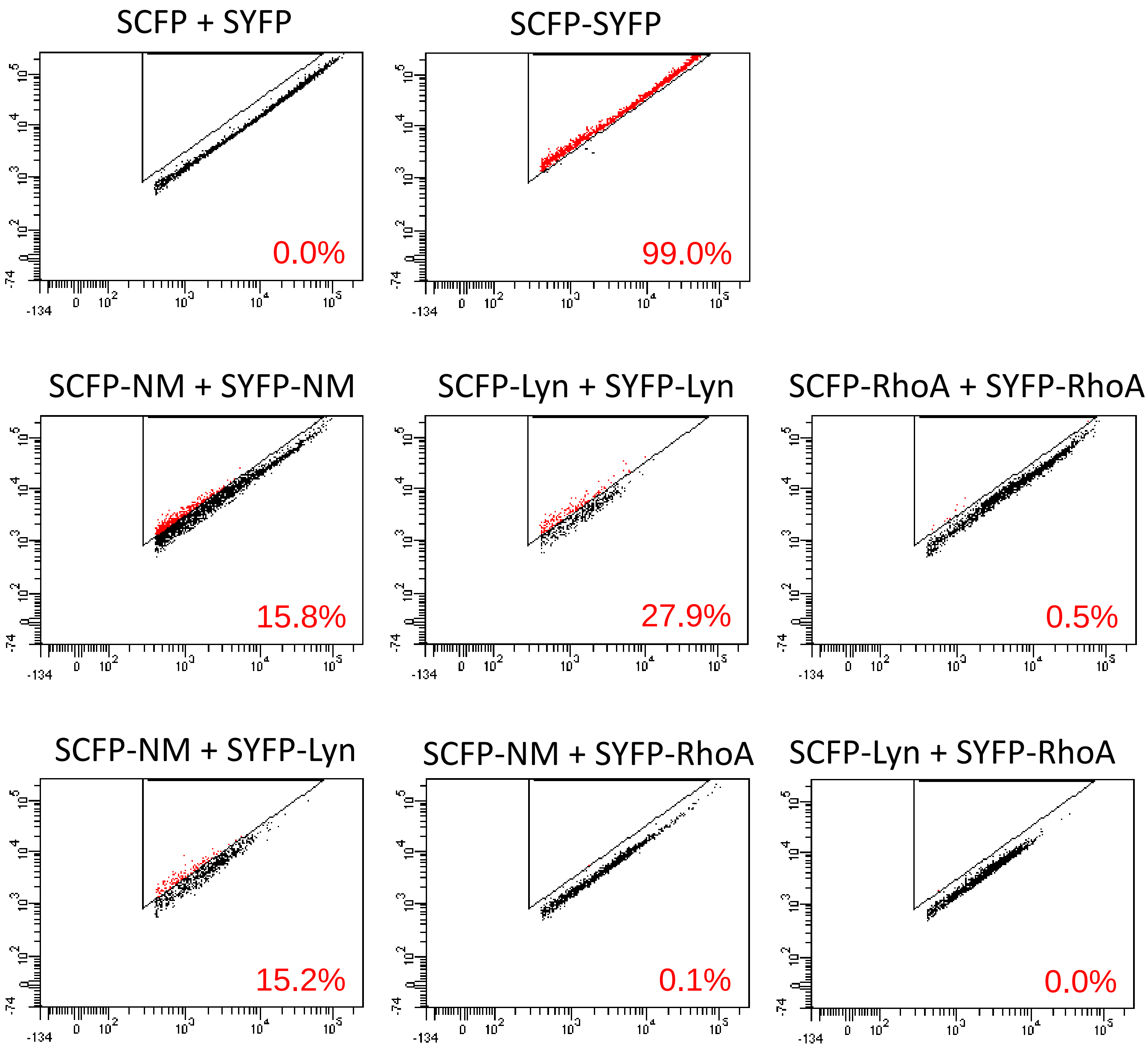

Suppl. Figure 2. Representative primary FACS-FRET plots complementing Figure 1D. Plots showing FRET vs. CFP signals and the gate used for evaluation of FRET+ cells are shown for individual samples used in figure 1D. The proteins coexpressed in 293T cells are indicated on top. FRET-positive cells are colored red and the percentage of FRET-positive cells is indicated.

Suppl. Fig. 3. **Winkler *et al.***

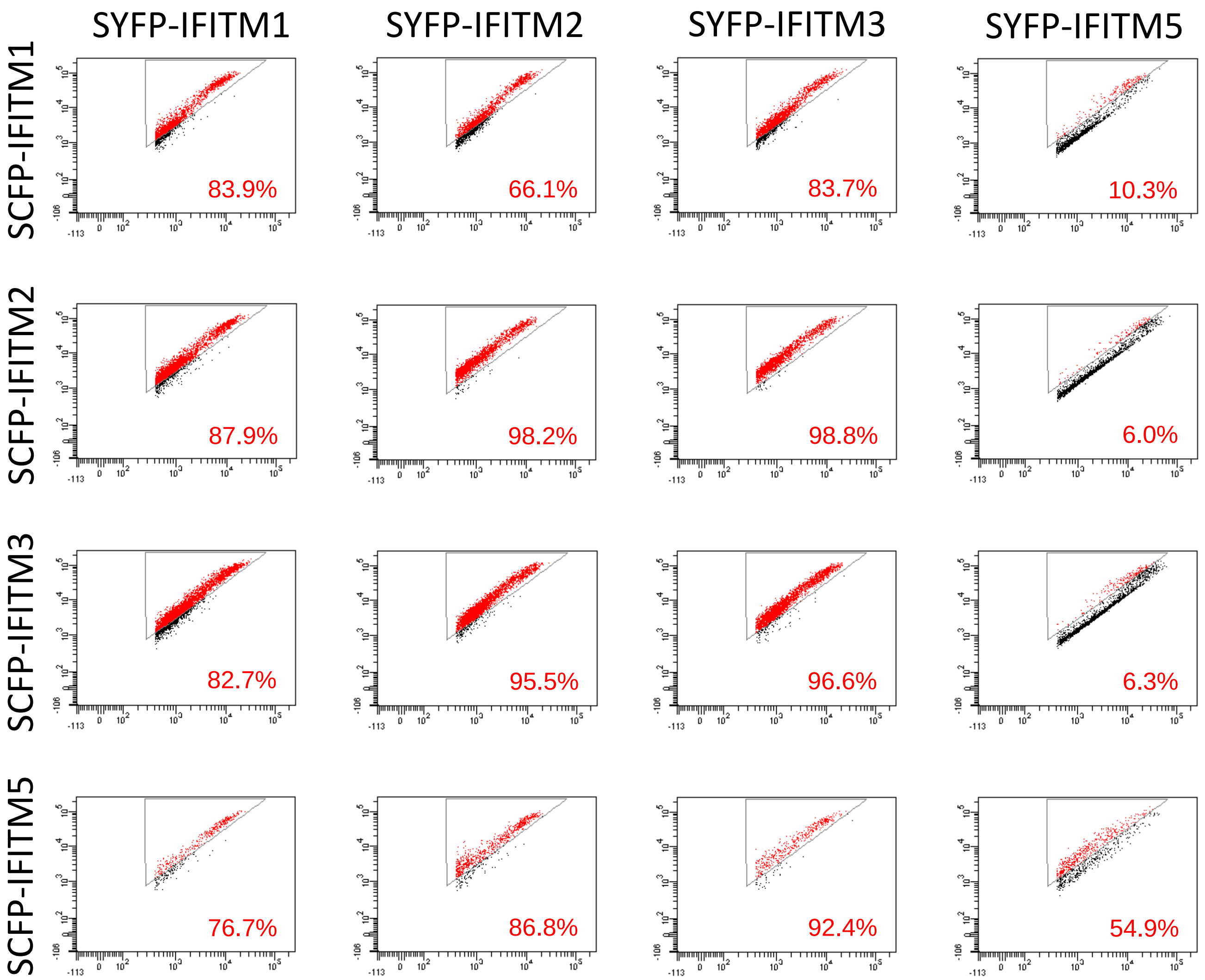

Suppl. Figure 3. Representative primary FACS-FRET plots complementing Figure 2C. Plots showing FRET vs. CFP signals and the gate used for evaluation of FRET-positive cells are shown for individual samples used in figure 2C. The plots are arranged as an array with the proteins coexpressed indicated on the left and on top. FRET-positive cells are colored red and the percentage of FRET-positive cells is indicated.

Suppl. Fig. 4. **Winkler *et al.***

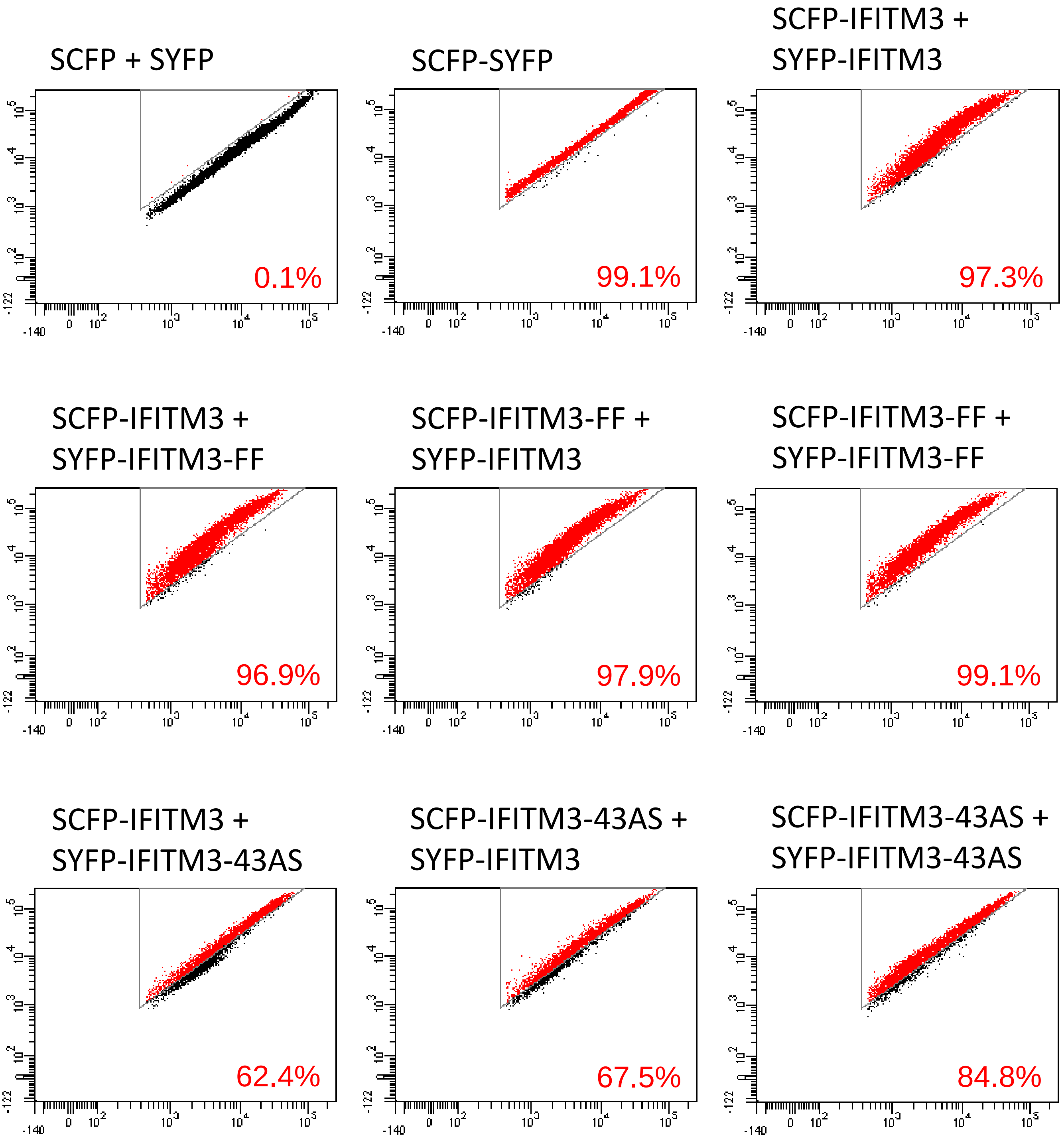

Suppl. Figure 4. Representative primary FACS-FRET plots complementing Figure 3E. Plots showing FRET vs. CFP signals and the gate used for evaluation of FRET-positive cells are shown for individual samples used in figure 3E. The proteins coexpressed in 293T cells are indicated on top. FRET-positive cells are colored red and the percentage of FRET-positive cells is given.
